# Athlete perceptions of flavored, menthol-enhanced energy gels ingested prior to endurance exercise in the heat

**DOI:** 10.1080/15502783.2022.2117995

**Published:** 2022-11-28

**Authors:** Roxanne M Vogel, Megan LR Ross, Christian Swann, Jessica E Rothwell, Christopher J Stevens

**Affiliations:** a Southern Cross University, Discipline of Sport and Exercise Science, Coffs Harbour, Australia; b GU Energy Labs, Berkeley, CA, USA; c Australian Catholic University, Melbourne, Australia; d Athletics Australia, Melbourne, Australia; e Victorian Institute of Sport, Australia

**Keywords:** Endurance, cooling, thermal perception, supplement, mint

## Abstract

**Background:**

L-menthol evokes a cooling sensation by activating cold sensing cation channels. Menthol-enhanced fluids can be ergogenic during exercise in the heat by improving thermal perception; hence, the addition of menthol to energy gels may benefit athletes. Previously, unflavored menthol gels were deemed acceptable at 0.1% concentration, but no research has been undertaken on menthol gels with additional flavoring. Therefore, we determined athlete perceptions of flavored energy gels with different menthol concentrations.

**Methods:**

With a randomized, crossover, double-blind, placebo-controlled design, 27 athletes (34.8 ± 6.7 y, 9 females) ingested an energy gel with either 0.1%, 0.3%, 0.5%, or 0.7% menthol concentration, or a non-menthol, flavor-matched placebo (CON), on separate occasions before outdoor exercise. Gels were rated for cooling sensation, irritation, flavor, and overall experience on 100-point sensory and hedonic labeled magnitude scales. The duration of any cooling sensation was also reported.

**Results:**

All menthol gels delivered a greater cooling sensation compared to CON (7.4 ± 8.1 AU) with a significantly greater response for 0.7% (59.9 ± 20.5 AU) and 0.5% (57.7 ± 21.8 AU), compared to all others. Irritation was higher for all menthol gels compared to CON (3.4 ± 7.2 AU) and for 0.7% compared to 0.1% (31.1 ± 31.0 vs. 16.3 ± 21.0 AU, p = 0.041), with none rated above a ‘mild-moderate’ intensity. The menthol gels delivered a significantly longer cooling sensation duration (12.3-19.6 min) versus CON (2.2 ± 4.8 min) with no difference between menthol gels.

**Conclusion:**

A flavored menthol energy gel at 0.1–0.7% concentration provides a cooling sensation for athletes when ingested before exercise. The 0.5% concentration is recommended to maximize the cooling sensation whilst minimizing irritation.

## 1. Introduction

Hot environmental conditions are known to impair endurance exercise performance, increase cardiovascular strain, and raise the risk of heat-related illness [1,2]. To overcome some of these issues, researchers have investigated various cooling strategies to improve endurance performance in the heat [e.g. 3]. Acute cooling strategies are beneficial to athletes immediately before and during exercise to attenuate the rise in core body temperature, improve thermal comfort, or enhance thermal tolerance. Many of these strategies have relied on internal and external thermal cooling – by means of lowering physical body temperature – such as cold baths, ice towels or garments applied to the athlete, or fanning/dousing athletes with cool mist or water [4]. More recently, L-menthol has been identified as an acute, non-thermal cooling method that can be applied either topically or internally before and during exercise [5]. L-menthol (menthol), which is derived of the *Mentha* plant species, including peppermint and corn mint, is widely used as a flavoring ingredient in oral hygiene products, gum, cough drops, and analgesic creams owing to its refreshing aroma and pleasant cooling sensation [6]. Menthol stimulates the transient receptor potential melastatin-8 (TRPM8) channel, the primary receptor for cold stimuli (<28°C) in thermosensitive neurons, which evoke a cooling sensation without altering the physical body temperature [7].

The internal (oral) application of menthol can be ergogenic for endurance exercise performed in warm-hot environments [5,8,9]. Performance in time to exhaustion and time trial efforts have resulted in improvements ranging from 2.3-9% among cyclists [10–12] and runners [13,14]. Studies involving the oral application of menthol have typically utilized a mouth rinse or beverage formulated at 0.01-0.1% menthol concentration administered repeatedly throughout (i.e. every 10 min) or once near the end of exercise trials [8,15,11,12]. Since menthol does not provide thermal cooling, these performance improvements cannot be explained by changes in body temperature. Rather, the mechanism of performance improvement is attributed to temporary adjustments in perception of temperature and ventilation, and potentially analgesia and arousal, resulting in a higher self-selected work rate [9].

The addition of menthol to commercially available sports nutrition products has garnered recent attention as a practical solution for athletes competing in the heat. A study in trained male cyclists found that a menthol-enhanced sports drink did not produce superior time to exhaustion performance results compared to a standard sports drink [16]. The authors speculated that a 0.01% menthol concentration might not be strong enough to have an ergogenic effect, or that it may have been overshadowed by the benefits of carbohydrate in the drinks. Energy gels are another widely used practical nutrient delivery form for athletes. Previous work from our laboratory conducted on elite endurance athletes demonstrated that an energy gel with a menthol additive was acceptable at a 0.1% concentration and provided a significant cooling effect compared to a non-menthol, flavor-matched placebo gel [17]. At 0.5% concentration, the menthol gel produced a greater cooling effect than 0.1%; however, the overall experience was significantly lower, and feedback indicated that the mint flavor was ‘*too strong’* for a majority of the participants surveyed (i.e. 34/40). The addition of sweeteners, carbohydrates, or other flavors to menthol-containing food and beverages can influence the perception of mint flavor and hence the overall sensory experience of these products [18,19]. For instance, the addition of sugar enhances the perception of menthol in mint-flavored beverages [18] and the inclusion of certain starches can delay the release kinetics of menthol in the presence of digestive enzymes [19]. As such, the current study sought to optimize a menthol-enhanced energy gel with additional flavoring, including natural citrus flavor, to identify whether the new formulation would increase the tolerability of gels with higher (i.e. >0.1%) menthol concentration. A secondary aim of the study was to determine the duration of the perceived cooling effect, if any, to inform future menthol gel administration protocols. We hypothesized that compared to placebo, consuming the optimized menthol gels would elicit higher ratings of perceived cooling sensation intensity and cooling duration.

## 2. Methods

### Experimental design

With a randomized, crossover, and double-blind placebo-controlled design, participants ingested either a flavored energy gel with a menthol additive at four different concentrations (i.e. 0.1%, 0.3%, 0.5%, or 0.7 %), or a flavor-matched placebo without menthol (CON). Trials occurred on separate days during outdoor endurance training sessions with measures designed to determine gel acceptability, as well as the time course of any perceived cooling sensation. Approval for the study was provided by the Southern Cross University Human Research Ethics Committee (approval number: 2021/005), and written informed consent was obtained from participants in accordance with the Declaration of Helsinki.

### Participants

Participants included 27 competitive, endurance-trained athletes (34.8 ± 6.7 y, BMI: 21.7 ± 1.6 kg·m^−2^, 9 females) including racewalkers (n = 4), triathletes (n = 3), and runners (n = 20). Self-reported training age was 12.8 ± 7.0 y and weekly running/racewalking volume was 10.0 ± 4.4 h. Inclusion criteria stipulated that athletes were: (a) aged between 18-40 y, (b) healthy, as assessed by the Exercise & Sports Science Australia adult pre-exercise screening system, (c) endurance-trained (i.e. consistently performing endurance exercise >30 min/day, at least three d/week over the previous three months). Participants were required to be injury free and capable of running/racewalking continuously for up to 60 min. Participants were excluded if they met any of the following criteria: (a) presence of anosmia or dysgeusia, (b) used any medicine or supplements that may have significantly affected participant safety (e.g. thermogenic supplements, amphetamine-based medications, diuretics), (c) had a known allergy or intolerance to any of the ingredients contained in the energy gels, (d) had a history of heat-related illness. Several participants were professional and amateur-elite athletes recruited through national sporting organizations and sport clubs, including six Olympic and World Championship athletes from Australia and the USA. Recruitment and data collection occurred both in Australia (January-March 2021) and the USA (June-August 2021) to correspond with the warmer months in each respective locale.

### Energy gels

The energy gels were produced in a commercial laboratory (GU Energy Labs, Berkeley, CA). A standard commercial energy gel base was produced using the following ingredients: maltodextrin, water, fructose, L-leucine, sodium citrate, medium-chain triglycerides, sea salt, potassium citrate, citric acid, calcium carbonate, L-valine, gellan gum, L-isoleucine, sodium benzoate (preservative), potassium sorbate (preservative), and natural citrus flavor. This base was used to create the five experimental gels. Four of the gel batches were mixed with natural L-menthol (Sigma-Aldrich, St. Louis, MO) at four different concentrations (i.e. 0.1%, 0.3%, 0.5%, or 0.7%). These concentrations were determined based on previous work comparing menthol gels with low (0.1%) and high (0.5%) concentrations to estimate what would be acceptable and preferable [17]. The addition of natural citrus flavor was novel to the menthol/control gel formulation used in the current study. The control gel consisted of the base plus a natural mint flavor derived from spearmint (*M. spicata*) devoid of the L-menthol isomer (Virginia Dare, Brooklyn, NY), which had a similar minty taste and aroma without the cooling action specific to L-menthol [20]. The gels were placed into 16 g single-use packets by an independent collaborator not involved with data collection. Each packet was labeled with a 3-digit numeric code to assist with the double-blind delivery. The samples were approved to be free from any 2020 World Anti-Doping Agency (WADA)-prohibited substances detected via independent evaluation (Human and Supplement Testing Australia, Flemington, Australia; LGC Sciences, Lexington, KY, USA) prior to athlete allocation.

### Procedure

Prior to all experimental trials, participants were instructed to avoid alcohol and strenuous exercise for 24 h, and to consume the same standardized pre-exercise meal (of their choice) two hours prior to the session. They were instructed to consume 500 mL of water one hour prior to exercise to promote adequate hydration. To avoid any desensitization effects with other menthol-containing products, participants were advised to avoid oral hygiene products (i.e. toothpaste, mouthwash) and mint-containing foods (e.g. mint tea, chewing gum, lozenges) for at least 2 h before each trial, as these have been known to reduce oral chemesthetic sensitivity for 60 minutes [21]. Adherence to these procedures was confirmed by participants prior to the collection of any survey responses.

Participants were provided with one 16 g sample of each of the five energy gels for consumption before running/racewalking sessions undertaken on separate days, with at least 72 h between trials. A Latin square design was used to randomly assign and counterbalance each participant to a trial order sequence. Testing sessions were conducted during the warmer months in the participants’ locale during the afternoon, to coincide with the highest ambient temperatures of the day. Participants recorded the outdoor temperature, windspeed, and relative humidity at the beginning of each session according to the current local weather forecast provided by either the Bureau of Meteorology (http://www.bom.gov.au/) in Australia or the National Weather Service (https://www.weather.gov/) in the USA. In the presence of a nonparticipating adult, participants ingested the entire gel sachet, swishing the contents around the mouth for ~5 s, followed by the ingestion of 150 mL of tepid water, 15 min before commencing exercise. No researchers were present due to Covid-19 restrictions; however, participants were asked to record the time of day they consumed the gel sachet to assess compliance. Participants were advised to consume the gels at the same time of day during each trial to avoid any potential diurnal effect on taste perception. Running and racewalking sessions lasting approximately 45 min were prescribed at a moderate-vigorous intensity [i.e. 13-15 RPE [22], or 70-85% of current-at-the-time maximum heart rate]. Exercise intensity was verified by the participants wearing a heart rate monitor. If no current, lab-verified maximal heart rate value was available, an age-based maximal heart rate was estimated using the equation of Fox [23]. The duration of the cooling sensation (if any) was timed using a personal watch or smartphone and recorded by the participant.

Immediately after each training session, participants completed an online questionnaire to assess perceptions of: (a) flavor intensity, (b) irritation, and (c) cooling sensation, via 100-point labeled magnitude scales anchored on either end by the words ‘none/absent’ corresponding to zero and ‘strongest imaginable’ corresponding to 100 arbitrary units (AU) [24-26]. Subjective assessments of overall experience and overall flavor were rated via general hedonic labeled magnitude scales, anchored on either end by ‘extremely dislike’ and ‘extremely like’ at zero and 100 AU, respectively. Gastrointestinal (GI) symptoms were measured with a modified Visual Analog Scale (VAS) with zero indicating no symptoms at all and 10 indicating ‘extremely bad symptoms’ [27]. Upper and lower GI symptoms evaluated were heartburn, nausea, bloating, belching, stomach pain, urge to regurgitate/regurgitation, lower abdominal pain/bloating, urge to defecate, flatulence, and cramp/stitch. Finally, participants recorded the total distance (km), duration (min), and average heart rate (derived from GPS tracking and heart rate monitor recordings, respectively) during their training sessions. Other than the pre-exercise 150 mL water, no other gels, foods, or beverages were consumed during the sessions. As data collection occurred during the COVID-19 pandemic (January-September 2021), participants and researchers had no physical contact, and all materials were mailed to participants. Athlete training schedules were not directly impacted by the pandemic, and all participants were able to train freely during this time. Data were collected exclusively via online survey (Qualtrics, Seattle, WA, USA).

### Statistical analysis

Data were analyzed via repeated-measures ANOVA with pairwise comparisons performed using a Bonferroni correction for multiple comparisons. To determine any potential confounding effect of ambient conditions (e.g. temperature, relative humidity), heat index was calculated using the equation of Rothfusz [28] and used as a covariate for a subsequent repeated-measures ANCOVA on primary outcome measures. Normality was assessed by Shapiro-Wilk tests and sphericity was assessed using Mauchly’s test of sphericity. When sphericity could not be assumed, a Greenhouse–Geisser correction was applied. Partial eta squared (η^2^) is presented as an estimate of effect size, and interpreted as: small (η^2^ = 0.01), medium (η^2^ = 0.06), and large (η^2^ = 0.14) effects [29]. All analyses were carried out using SPSS Statistics (version 28.0; IBM Corp., Armonk, NY). Data are presented as mean ± standard deviation.

## 3. Results

Trials were undertaken near midday (*M* 12:19 PM, range 04:30-20:00 h), and ambient conditions were not significantly different between trials (27.5 ± 6.8°C, 58.2 ± 23.2 % RH, 10.3 ± 5.7 km·h^−1^ windspeed). Average run-derived variables across trials were not significantly different, with runs lasting approximately one hour in duration (59.7 ± 25.0 min), covering 11.0 ± 4.4 km distance, at heart rate of 149.6 ± 17.3 bpm.

**Figure 1.**
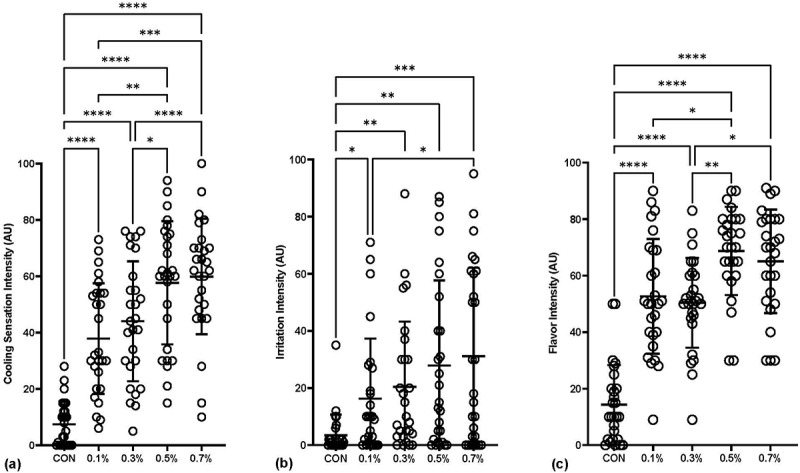
Ratings of (a) Cooling sensation intensity, (b) irritation intensity, and (c) flavor intensity. Results presented as individual responses (circles) and mean ± SD. AU = arbitrary units. Level of significance indicated as *p < 0.05, **p < 0.01, ***p < 0.001, ****p < 0.0001.

Ratings of cooling sensation intensity are illustrated in Figure 1a. A significant main effect for cooling sensation intensity was observed *F*(4, 104) = 52.90, *p* < .001, partial η^2^ = 0.67. All menthol gels successfully delivered a greater cooling sensation compared to CON (7.4 ± 8.1 AU) with a significantly greater response for 0.7% (59.9 ± 20.5 AU) and 0.5% (57.7 ± 21.8 AU), compared to all others, which were both rated ‘moderate-strong’ for intensity. When measures of cooling sensation intensity were analyzed by ANCOVA with heat index as the covariate, outcomes were consistent with the ANOVA analysis (*p* < .001).

Ratings of irritation intensity are illustrated in Figure 1b. A significant main effect for irritation intensity was observed *F*(4, 104) = 11.19, *p* < .001, partial η^2^ = 0.30. All menthol gels were rated higher for irritation intensity compared to CON, and 0.7% was higher than 0.1%. No gel was rated as higher than ‘mild-moderate’ on the irritation intensity scale (i.e. a value of approximately 30 on a zero to 100 scale). When measures of irritation intensity were analyzed by ANCOVA with heat index as the covariate, outcomes were consistent with the ANOVA analysis (*p* < .001).

Ratings of flavor intensity are illustrated in Figure 1c. A significant main effect for flavor intensity was observed *F*(4, 104) = 44.44, *p* < .001, partial η^2^ = 0.63. All menthol gels were rated as having a greater flavor intensity compared to CON with a significantly greater response for 0.5% (68.7 ± 15.6 AU) than all other conditions, except for 0.7%. When measures of flavor intensity were analyzed by ANCOVA with heat index as the covariate, outcomes were consistent with the ANOVA analysis (*p* < .001).

**Figure 2.**
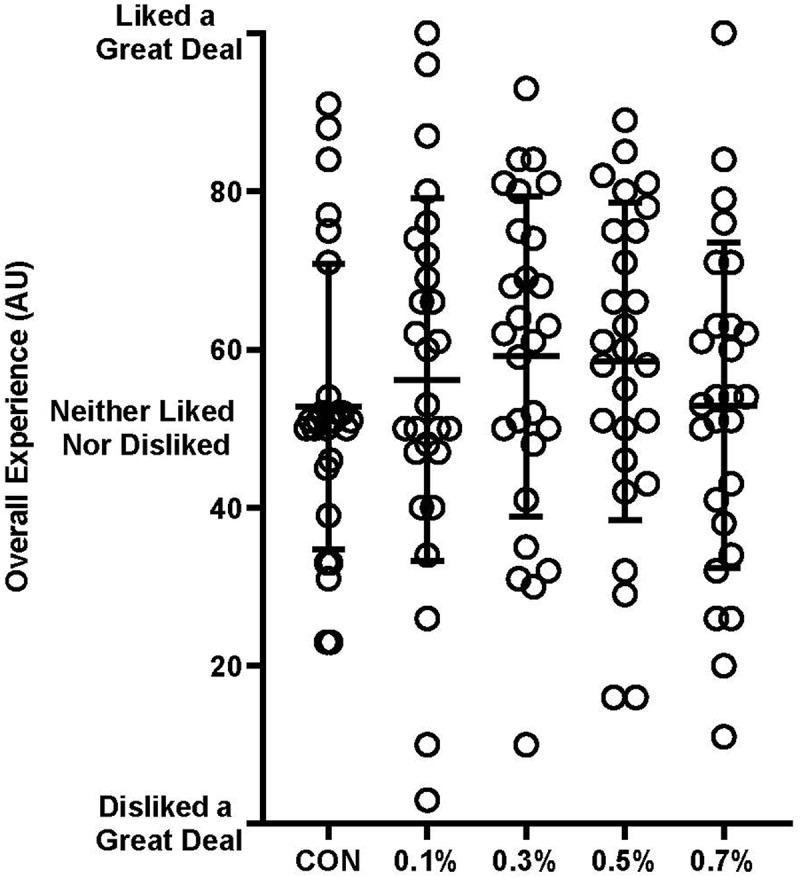
Ratings of overall experience. Results presented as individual responses (circles) and mean ± SD. AU = arbitrary units.

Ratings of overall experience are illustrated in Figure 2. No significant difference was observed between conditions *F*(4, 104) = 0.82, *p* = .514. Similarly, no difference was observed between overall flavor experience, which although violated the assumption of sphericity (χ^2^(4) = 46.12, *p* < .001), did not reach significance (*F*(1.985, 51.619) = .70, *p* = .597) when a Greenhouse-Geisser correction was applied with Epsilon (ε) = 0.496.

No significant differences between conditions were observed for any GI symptom (heartburn, nausea, bloating, belching, stomach pain, urge to regurgitate/regurgitation, lower abdominal pain/bloating, urge to defecate, flatulence, cramp/stitch), and all were rated below a value of 1, indicative of ‘none’ to ‘very mild’ symptoms on the VAS scale. GI symptom data are available as supplemental files upon reasonable request.

**Figure 3.**
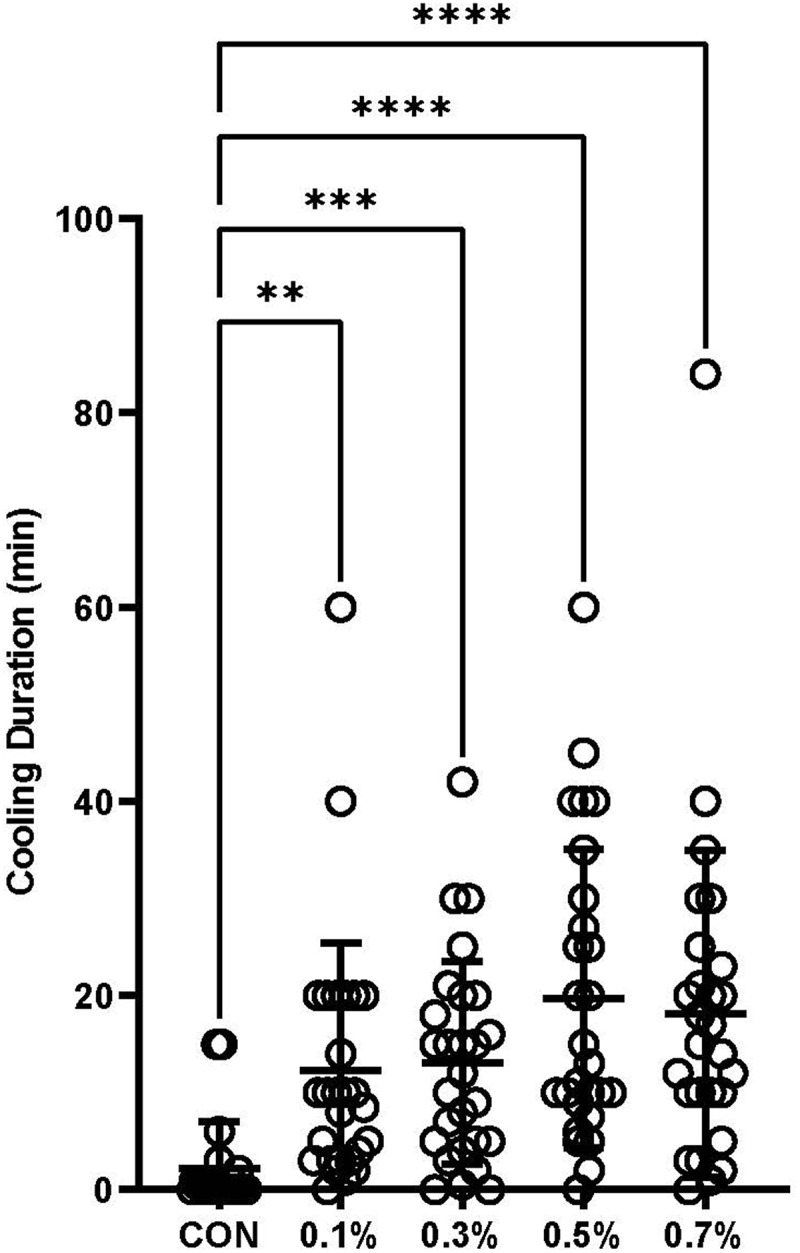
Reported duration of cooling sensation in minutes. Results presented as individual responses (circles) and mean ± SD. Level of significance indicated as *p < 0.05, **p < 0.01, ***p < 0.001, ****p < 0.0001.

Reported duration of cooling sensation is illustrated in Figure 3. Mauchly’s test of sphericity indicated that the assumption of sphericity had been violated χ2(9) = 17.57, p = .041. Epsilon (ε) was calculated as 0.742, and a Greenhouse-Geisser correction applied. With this correction, the gel samples elicited statistically significant differences in cooling duration, *F*(2.968, 77.177) = 11.01, p < .001, partial η2 = .30. All four menthol gels successfully delivered a significantly longer cooling duration (range: 12.3-19.6 min) compared to CON (2.2 ± 4.8 min) with no significant difference between menthol gels. When measures of cooling duration were analysed by ANCOVA with heat index as the covariate, outcomes were consistent with the ANOVA analysis (*p* < .001).

## 4. Discussion

The major findings of the current study are that a flavored, 0.1-0.7% menthol-enhanced carbohydrate energy gel produces a significantly greater perceptual cooling intensity and duration compared to a non-menthol flavor-matched control. Specifically, the 0.5% menthol concentration gel was the most effective for maximizing the intensity and duration of the cooling effect whilst minimizing irritation. Earlier work indicated that an unflavored menthol-enhanced gel at a 0.5% concentration provided a significant cooling effect but a lower overall experience owing to the mint flavor being ‘*too strong’* compared to a 0.1% menthol gel [17]. The addition of a natural citrus flavor to the formulation appears to increase the tolerability to a higher menthol concentration, potentially by masking the intensity of menthol/mint flavor which can impart a bitter taste on the tongue [30], and hence improving overall palatability. A 0.5% concentration is higher than the current recommendations from an international consensus statement on the use of menthol for athletes [5] and the Australian Institute of Sport’s supplement framework factsheet on menthol as an ergogenic aid [31] which both suggest a concentration of 0.01-0.05% for menthol beverages and mouth rinses, based on the available literature at the time these recommendations were made. It is also higher than other commercially available menthol-based sports nutrition products on the market, including an energy gel and drink mix containing 0.01% menthol (Turbo+, Science in Sport, London, UK).

All four menthol-enhanced gels outperformed CON for cooling sensation and duration, consistent with prior research indicating that unflavored, menthol-enhanced products produce a noticeable cooling sensation compared to placebo [17]; however, the duration of the cooling effect was not measured in the previous study. Thus, the observed ‘cooling’ duration for the menthol gels (range: 12.3-19.6 min) provides novel information for practitioners looking to implement these products with their athletes prior to training and competition as part of their performance nutrition planning. It is not currently known what the optimal interval between menthol oral exposures is, nor the ideal duration of oral contact, but evidence suggests possible desensitizing with repeated exposures [32,33]. Therefore, a single exposure administered late in an endurance effort may be sufficient to improve performance, as previously reported by Jeffries and colleagues [15).

There was no difference in ratings of overall experience or flavor among any of the gels, despite higher irritation ratings for the menthol-enhanced samples. This suggests that although there was a noticeable ‘tingling/burning’ sensation, the effect was not sufficient to negatively impact the athletes’ overall experience nor the overall enjoyment of the gel flavor. Conversely, it could be said that the higher concentration gels (i.e. 0.5% and 0.7%) did not outperform the lower concentration gels (i.e. CON, 0.1%, and 0.3%) in terms of the athletes’ overall experience. Future formulations may attempt to improve upon the flavor and hedonic experience of menthol-enhanced sports nutrition products. It has been demonstrated that adjusting flavoring elements and sweeteners can augment the sensory experience of mint-flavored beverages [18], and hence, there are many permutations of these elements that could be experimented with to optimize the user experience of menthol energy gels. It has also been shown that the dilution method of menthol crystals into solution can alter the subsequent thermal perception of the formulation [34]. It is possible, given the novel formula of the gels used in the current study, that the consistency, inclusion of flavoring agents, starches, and sugars, and/or the method of menthol dilution may alter perceptions of the gel, and that a different formulation or method of preparation may change the overall sensory experience.

Gastrointestinal issues surveyed in the current study were very mild (i.e. < 1 on a 0-10 scale, with zero indicating ‘none at all’) and were not different between gels. Menthol is known to alleviate symptoms related to irritable bowel syndrome and functional dyspepsia, and help relax the smooth muscle of the GI tract [35]. Energy gels are specifically formulated for consumption during exercise, and are commonly provided by race directors for competitors during organized endurance events. The base formula of the gels used in the current study is similar to a commercially available product (GU Energy Gel, GU Energy Labs, Berkeley, CA) which has been used extensively by athletes during training and competition, and has been evaluated under laboratory conditions [36]. Moreover, all participants surveyed were familiar with consuming energy gels prior to their enrollment in the study and indicated they had no food allergies or sensitivities to any of the ingredients in the gel samples.

Strengths of the current study include a sample comprising both male and female athletes, the use of a true (i.e. flavor-matched) placebo for the CON trial, and an ecologically valid design with runners and racewalkers consuming the gels before self-paced running in warm/humid outdoor conditions. Further, the menthol gel formula is a commercially viable one that could be used by athletes of all levels in sanctioned endurance events. Indeed, following data collection, some of the participants in the present study went on to request and use additional samples of the menthol enhanced gels during the 2020 Tokyo Olympics as part of their performance nutrition plans (4th author, personal communication, 25 November 2021).

Limitations of the study include a small, heterogenous sample size comprising both elite and highly-trained endurance athletes, and relatively moderate – albeit humid – ambient conditions during the running sessions (28°C, 58 % RH). Another potential limitation is that an unflavored menthol gel was not included, so no direct comparisons can be drawn between perceptions of flavored versus unflavored menthol gels. Also, the intensity of the exercise sessions was moderately hard (RPE 13-15), which reflects training but not necessarily the intensity of shorter competitions. Finally, participants were not asked at the time of survey whether they were experiencing any loss of taste or smell, which could have impacted perceptual responses. Future research in this area should include a larger female cohort, and potentially a homogenous sample of either all-elite or all recreationally trained athletes, or a cohort of each so that comparisons may be drawn. Research among females and elites employing menthol cooling is currently limited [5], although recent work has established the potential for sex-based differences in the perceptual and subsequent thermo-behavioral effects of this strategy [12]. More data are required to confirm these findings. Finally, it remains to be seen what the best application strategy is for menthol-based gels. Considerations include timing, duration of exposure to the oral cavity (i.e. ‘swilling’ time), single versus repeated dosing, dosage amount, temperature of the product (i.e. chilled or room temperature), and the temperature of any subsequent ingested fluids, which could further potentiate the cooling sensation of the menthol gel.

The results of this study may be used by coaches, sports nutritionists/dietitians, and athletes when determining which acute cooling methods to implement prior to training or competition in the heat. As GI issues are known to negatively impact endurance performance, a menthol-enhanced energy gel that doesn’t provoke GI problems and provides a cooling sensation when consumed before exercise could be a practical and effective strategy for consideration.

## Conclusion

The results of the present study demonstrate that a flavored, menthol-enhanced energy gel at 0.5% menthol concentration is acceptable and provides a cooling effect lasting approximately 20 min when taken prior to exercise in the heat. As such, menthol gels may be used to help athletes feel cooler without causing GI distress, and could be considered for use in warm conditions. These findings are potentially relevant for future sports nutrition product development to create a suitable commercial product for athletes.
